# Cryo-EM structure of PML RBCC dimer reveals CC-mediated octopus-like nuclear body assembly mechanism

**DOI:** 10.1038/s41421-024-00735-3

**Published:** 2024-11-25

**Authors:** Yangxia Tan, Jiawei Li, Shiyan Zhang, Yonglei Zhang, Zhiyi Zhuo, Xiaodan Ma, Yue Yin, Yanling Jiang, Yao Cong, Guoyu Meng

**Affiliations:** 1 Shanghai Institute of Hematology, State Key Laboratory of Medical Genomics, National Research Center for Translational Medicine, Rui-Jin Hospital, School of Medicine and School of Life Sciences and Biotechnology, Shanghai Jiao Tong University, Shanghai, China; 2 Key Laboratory of RNA Innovation, Science and Engineering, Shanghai Institute of Biochemistry and Cell Biology, Center for Excellence in Molecular Cell Science, Chinese Academy of Sciences, Shanghai, China; 3 University of Chinese Academy of Sciences, Beijing, China; 4 Department of Geriatrics and Medical Center on Aging, Rui-Jin Hospital, Shanghai Jiao Tong University School of Medicine, Shanghai, China; 5 State Key Laboratory of Pathogenesis, Prevention and Treatment of High Incidence Diseases in Central Asia, First Affiliated Hospital of Xinjiang Medical University, Urumqi, Xinjiang China; 6 National Facility for Protein Science in Shanghai, Shanghai Advanced Research Institute, Chinese Academy of Science, Shanghai, China; 7 Key Laboratory of Systems Health Science of Zhejiang Province, School of Life Science, Hangzhou Institute for Advanced Study, University of Chinese Academy of Sciences, Hangzhou, Zhejiang China

**Keywords:** PML bodies, Leukaemia, Cryoelectron microscopy

## Abstract

Promyelocytic leukemia protein (PML) nuclear bodies (NBs) are essential in regulating tumor suppression, antiviral response, inflammation, metabolism, aging, and other important life processes. The re-assembly of PML NBs might lead to an ~100% cure of acute promyelocytic leukemia. However, until now, the molecular mechanism underpinning PML NB biogenesis remains elusive due to the lack of structural information. In this study, we present the cryo-electron microscopy (cryo-EM) structure of the PML dimer at an overall resolution of 5.3 Å, encompassing the RING, B-box1/2 and part of the coiled-coil (RBCC) domains. The integrated approach, combining crosslinking and mass spectrometry (XL-MS) and functional analyses, enabled us to observe a unique folding event within the RBCC domains. The RING and B-box1/2 domains fold around the α3 helix, and the α6 helix serves as a pivotal interface for PML dimerization. More importantly, further characterizations of the cryo-EM structure in conjugation with AlphaFold2 prediction, XL-MS, and NB formation assays, help unveil an unprecedented octopus-like mechanism in NB assembly, wherein each CC helix of a PML dimer (PML dimer A) interacts with a CC helix from a neighboring PML dimer (PML dimer B) in an anti-parallel configuration, ultimately leading to the formation of a 2 µm membrane-less subcellular organelle.

## Introduction

Stress in the nucleus is a significant scientific issue that has garnered considerable attention in recent years. The nucleus is not only the control center of the cell but also a highly dynamic and sensitive organelle that responds to various stressors, including DNA damage, oxidative stress, and changes in temperature or pH. Membrane-less organelles (MLOs) play a crucial role in nuclear stress^1–3^. MLOs are dynamic, membrane-less structures that form and dissolve through phase separation of proteins and nucleic acids. MLOs, such as nuclear speckles, nucleoli, and stress granules, play crucial roles in the organization and regulation of nuclear processes^4,5^. In the context of nuclear stress, MLOs serve as hubs for coordinating the cellular response. Currently, our understanding of MLO assembly and phase separation is limited. Promyelocytic leukemia protein (PML), belonging to the TRIM family, serves as a valuable model in this field.

TRIM proteins are a structurally conserved and rapidly evolving protein family that comprises over 80 distinct members^6^. They feature an N-terminal tripartite RBCC motif (i.e., RING (R), B-box1/2 (B), coiled-coil (CC)) and exhibit various structural variations at the C-terminal region^7,8^. The different functional domains of TRIMs have distinct roles. The RING and B-box domains are zinc-binding domains. The RING is present in most TRIM proteins and is known to possess E3 ubiquitin ligase activity^9^. Additionally, B-box dimerization/trimerization is also crucial. For instance, TRIM5α’s B-box trimerization is pivotal for the megaDalton polymerization required for virus recognition^10^. The CC domain can self-associate, enabling different TRIM proteins to form high-molecular weight complexes in vivo, facilitating the formation of homo- or hetero-oligomers^8^. The C-terminal domains of TRIMs are unique and often interact with specific proteins, thus achieving differentiated functions^11–13^. Despite the conserved RBCC domains, TRIM proteins regulate diverse biological processes such as intracellular signaling, immunity, transcription, autophagy, and carcinogenesis^14,15^. For example, TRIM21, TRIM28, TRIM55, and TRIM63 proteins act as regulators of autophagy^16–19^, whereas TRIM5, TRIM19, TRIM23, and TRIM25 exhibit viral inhibitory effects^20–23^. Additionally, TRIM24, TRIM28, and TRIM29 play key roles in preventing carcinogenesis and the progression of cancer^24–26^.

The PML protein, also known as TRIM19, was originally identified in 1960^27,28^. As of the time of this report, it has been documented to directly or indirectly interact with approximately more than 160 proteins^29^. For example, SP100 protein co-localizes with PML as a transcriptional regulator^30,31^, and PML can be modified by SUMO-1/2/3 at three major SUMO sites (K65, K160, and K490)^32^. Within the nucleus, this 70 kDa PML protein molecule has the capability to assemble into a super protein complex, known as PML nuclear bodies (NBs). Under fluorescence microscopy, PML NBs appear as MLOs^33^, which have two distinct features: (1) variable sizes ranging from 0.1 μm to 2 μm^34^, and (2) oil-like droplets enabling efficient protein interaction and dynamic re-assembly^35^. PML NBs participate in various important cellular pathways, including DNA damage repair^36–39^, transcriptional regulation^40–42^, self-renewal of stem cells^43^, regulation of p53 stability^44^, cell proliferation^45–47^, apoptosis^48,49^, and defense against viral infections^50,51^. Besides, partner proteins concentrated within NBs may depend on the SUMO-interacting motif (SIM) for their positioning^33,52,53^, making PML NB a central hub in cellular signaling pathways^44,54–59^. At present, more than 10 isoforms of the PML protein have been identified. The conserved RBCC domain, located in the PML N-terminal region, plays a crucial role in its polymerization activity and the formation of NB^60–62^. In our latest work, we reported the crystal structure of the PML RING tetramer, which is thought to be important for the recruitment of the SUMO E2 ligase UBC9^61^. Additionally, the PML B-box1 (B1) contributes to overall PML RBCC oligomerization. Nmer-like polymerization in this network concentrates K160, favoring essential poly-SUMOylation modification for acute promyelocytic leukemia (APL) leukemogenesis^60^. Recently, Wu and coworkers demonstrated that B-box2 (B2) is the main domain involved in the phase transition and proposed a hypothesis of liquid–liquid phase separation (LLPS)-driven PML NB biogenesis: PML–PML interaction may be primarily mediated by RBCC oligomerization^62^. Multimers of PML, influenced by local protein concentrations, post-translational modifications (PTMs), and partner protein recruitment, could assemble via LLPS, ultimately forming a remarkable subcellular organelle with variable sizes^62^. The assembly of PML NBs determines their functions; therefore, elucidating the underlying mechanism is of great physiological significance for exploring the roles. Despite the working hypothesis of LLPS-driven PML NB biogenesis, the overall PML-RBCC structure remains elusive, and the structure of PML CC domain has not been reported. To fully elucidate the assembly mechanism of PML NBs, obtaining the complete structural information on PML-RBCC is essential.

In this study, we report the cryo-EM structure of the PML_46–256_ dimer, which encompasses the entire RING-B1-B2 domains and a segment of the CC domain. This RBCC dimer structure reveals a previously unrecognized fold in the TRIM RBCC assembly. Unlike other TRIMs, the RING, B1 and B2 subdomains are folded together, centered around the PML α3 helix, resulting in a PML monomer. The dimeric interaction is mainly mediated by hydrophobic interactions among residues V123, F177, and L178. Surprisingly, based on the current structure, the two CC domains are positioned on the opposing sides of the dimer, extending away from the core of the PML dimer. This arrangement, in conjugation with further AlphaFold2-based prediction, crosslinking and mass spectrometry (XL-MS), and NB formation analysis, helps uncover a previously unrecognized CC-based octopus-like assembly step in PML NB biogenesis.

## Results

### PML_46–256_ forms a dimer revealed by cryo-EM and XL-MS

**Fig. 1:**
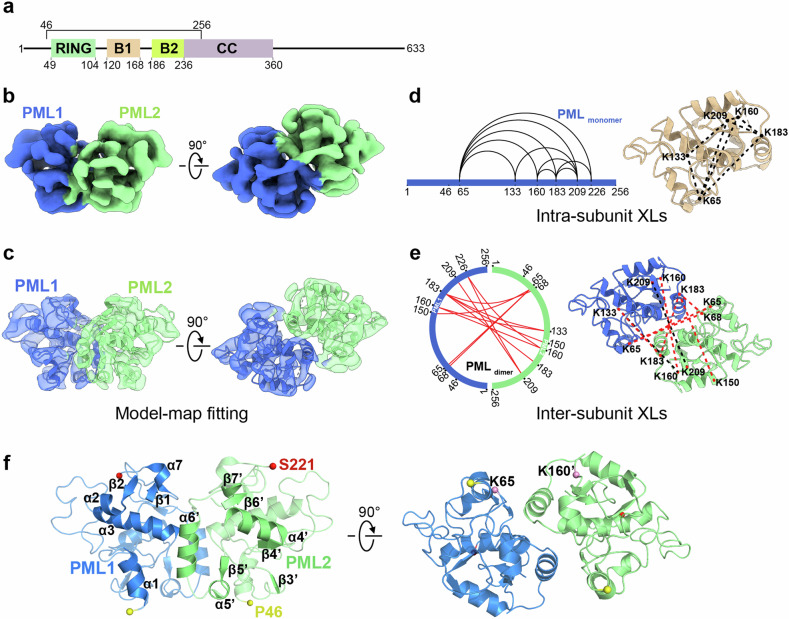
Cryo-EM characterization of the PML RBCC domain. **a** Depiction of full-length PML’s domain arrangement, highlighting the PML RBCC fragment (residues 46–256 used for structure determination). Different domains were depicted in distinct colors. **b** Cryo-EM density map of PML_46–256_ dimer. **c** Model-map fitting of the PML_46–256_ dimer. **d** XL-MS analysis of the MBP-PML_46–256_ monomer (left), with identified intra-subunit XLs also mapped on the PML_46–256_ monomer model and shown as black dotted lines (right). **e** XL-MS analysis of the MBP-PML_46–256_ dimer (left). Identified inter-subunit XLs were also mapped on the PML_46–256_ dimer model. Red and black dashed lines indicate XLs with Cα–Cα distances less than 35 Å and greater than 35 Å, respectively (right). We used the best E-value (1.00 × 10^–2^) and spec count of at least 2 as the threshold to remove XL-MS data with lower confidence. **f** Atomic model of the PML_46–256_ dimer, with each monomer colored in blue and green, and the secondary structure elements numbered as α1–α7 and β1–β7. The N-terminal P46 was marked as a yellow sphere, the C-terminal S221 as a red sphere, and the SUMO sites K65 and K160 as pink spheres.

For the past three decades, the purification of the full-length PML protein has posed significant challenges in the field. In our previous functional study, we managed to purify an extended N-terminal PML containing residues 1–256^60^. However, when this RBCC fragment was subjected to cryo-EM study, it exhibited a tendency to aggregate, resulting in substantial instability for cryo-EM visualization. In response to these challenges, we devised a different strategy. We omitted the N-terminal residues 1–45, which was predicted to be a flexible loop-like region (Fig. 1a). This PML_46–256_ fragment (~24 kDa) encompasses the complete RING, B1, and B2 domains, along with a segment of the CC domain (residues 236–256). Additionally, it includes the non-conserved helices situated within RBCC, which previously lacked structural information (Supplementary Fig. S1a–e). The resulting PML_46–256_ was then fused with an N-terminal maltose-binding protein (MBP) tag to enhance solubility and stability (Supplementary Fig. S1a), particularly during cryo-EM sample preparation (Supplementary Fig. S2a). Moreover, our initial cryo-EM visualization revealed that the PML_46–256_ particles in vitrified ice exhibited a problem of preferred orientation. To address this issue, we adopted the stage tilt strategy^63^, tilting the stage by 40°, which has been proven effective in various systems encountering similar problems^64,65^ (Supplementary Fig. S2b). After multiple rounds of 2D and 3D classification to eliminate heterogeneous and incomplete components, we determined the cryo-EM structure of PML_46–256_ at an overall resolution of 5.3 Å, with the core region slightly better resolved (~4.4 Å) (Fig. 1b; Supplementary Fig. S2c–f and Table S1). Interestingly, our structure disclosed that PML_46–256_ adopted a dimeric configuration (Fig. 1b), which could be confirmed by the reference-free 2D class averages (Fig. 1b; Supplementary Fig. S2b, c). We then built an atomic model for PML_46–256_ dimer (Fig. 1c) on the basis of the AlphaFold2^66,67^-predicted model, along with the previously reported crystal structures of the certain portions of the RING, B1, and B2 domains^60,61,68^, with constraints of the current cryo-EM map.

To confirm that the PML_46–256_ indeed adopts a dimeric configuration, we performed further XL-MS analysis on the MBP-PML_46–256_ monomer and dimer, using bis(sulfosuccinimidyl) suberate (BS3) as the crosslinker. BS3 consists of two *N*-hydroxysulfosuccinimide (NHS) ester groups at both ends, which react with primary amines to form stable amide bonds. The spacer arm distance of BS3 is ~11.4 Å. Considering the Lys side chains as well as backbone dynamics, it is usually assumed that Lys residues within a Cα–Cα distance of up to 35 Å for BS3 would be preferentially crosslinked^69,70^. The data derived from the monomer served as a baseline, representing intra-subunit crosslinks (XLs) (Fig. 1d; Supplementary Table S2). In the case of the dimer, we eliminated the intra-subunit XLs, and the remaining 12 XLs were presumed to represent the inter-subunit XLs between the two monomers (Fig. 1e; Supplementary Table S3). It was evident that the signals of the residues K65, K68, K160, K183, and K209 were frequently detected in XL-MS analysis (Supplementary Table S3), indicating that these residues were spatially close to each other (Fig. 1e). The presence of multiple inter-subunit XLs confirmed that the PML_46–256_ indeed adopts a dimeric configuration. Due to the dynamics of the MBP tag relative to the target PML fragment, we could not capture the MBP density in our final reconstruction. To rule out the possibility that the dimer configuration might be an artifact induced by MBP tag, we removed the MBP tag and performed the XL-MS analysis on the PML_46–256_ dimer fraction (Supplementary Fig. S1b, c and Table S4). Supportively, eight intermolecular XLs were consistently detected with or without MBP, suggesting that MBP might play little role in PML dimerization (Supplementary Fig. S1c and Table S4). Overall, almost all detected intra/inter-subunit XLs fit the 35 Å Cα–Cα distance restraint, suggesting a high quality of the cryo-EM map and model building.

In our cryo-EM model, each PML monomer comprises helices α1–α7 and beta strands β1–β7 (Fig. 1f; Supplementary Fig. S1e). Additionally, two SUMOylation sites, K65 and K160, are located on the outer surface of the dimeric PML_46–256_, giving ample space for PML SUMOylation (Fig. 1f). Prior to this cryo-EM structure, it was reported that RING, B1, and B2 could undergo self-oligomerization^60,61,68^. Considering this, we also examined the published RING/B1/B2 oligomerization-related residues in the PML_46–256_ dimer. Consistently, the residues reported on the RING/B1/B2 interaction interfaces were also found on the outer surface of the dimeric PML_46–256_ (Supplementary Fig. S1d). Despite the prediction that the CC domain would be a long helix, the density of the C-terminal CC residues 236–256 were not visible in this PML dimer. This observation suggests that the CC region might exhibit greater flexibility than previously anticipated (discussed later).

### PML monomer

**Fig. 2:**
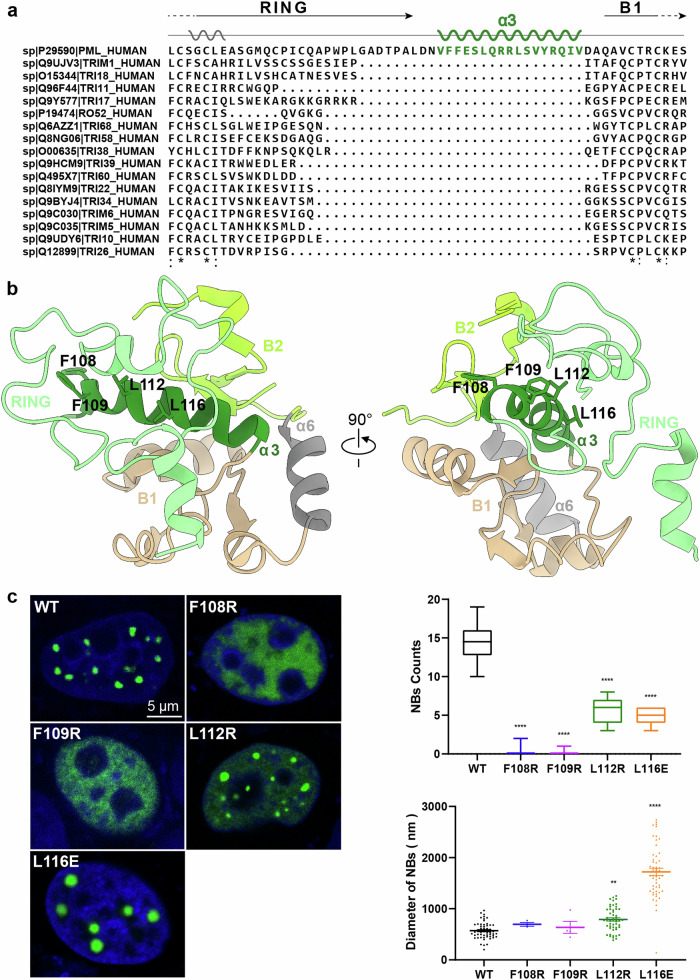
PML monomer. **a** Sequence alignment of the non-conserved region between the RING and B1 domains in human TRIM proteins. The α3 helix, which is unique to PML when compared with other TRIM proteins, was highlighted with dark green. **b** Interaction network of the α3 helix with the RING, B1, and B2 domains. α3 was depicted in dark green, RING in light green, B1 in tan, B2 in yellow green, and α6 in gray. **c** Fluorescence microscopy images of HeLa^*Pml−/−*^ cells expressing EGFP-PML, EGFP-PML_F108R_, EGFP-PML_F109R_, EGFP-PML_L112R_, and EGFP-PML_L116E_. Scale bar, 5 µm. Statistical analysis of PML NB formation was conducted. All experiments were performed in independent replicates, with NB counts calculated from ≥ 15 nuclei. Data are means ± SEM. **P < 0.01, ****P < 0.0001 (derived from ordinary one-way ANOVA test). All experiments/results displayed in the main figure were conducted with PML isoform IV.

Among all published TRIM RBCC structures^71–73^, the PML RBCC stands out. Prior to this work, most RBCC monomers appeared to have a loose architecture (Supplementary Fig. S3b), with the RING and B1/2 domains lying spatially apart from each other. This was also consistent with the result of AlphaFold2 prediction. By contrast, our cryo-EM map revealed a more compact structure, with the RING, B1 and B2 domains orientated in distinct topology (Supplementary Fig. S3a). Notably, the α3 helix, unique to PML (Fig. 2a), is at the central of the PML monomer. The RING, B1, and B2 domains are arranged surrounding the α3 helix and are distributed on the outer surface of the PML molecule. In this inner α3 helix, residues F108, F109, L112, and L116 form a hydrophobic core, intermittently mediating the engagement of the PML RING, B1 and B2, respectively (Fig. 2b).

To evaluate the significance of the hydrophobic core, we individually mutated the residues F108, F109, L112, and L116 of PML-IV to hydrophilic amino acids (i.e., Arg and Glu). Although fusion of a green fluorescent protein (GFP) at the PML N-terminus (GFP-PML) has frequently been used in the investigation of PML NB formation^60,61,74^, we also performed a control experiment of GFP alone (Supplementary Fig. S6c). The consistent results indicate that GFP-PML is effective for studying NB assembly in cells. We then utilized fluorescence confocal microscopy to check whether these alterations in the α3 helix could hinder the formation of PML NBs (Fig. 2c). As anticipated, the mutations F108R and F109R completely abolished PML NB formation (Fig. 2c). Mutations L112R and L116E led to larger but fewer NBs, accompanied by an increased level of diffused PML in the background (Fig. 2c). Of note, L116 is adjacent to the two consecutive Arg residues (i.e., R114 and R115); thus a Glu substitution was used to characterize this position (Fig. 2a, b). The findings suggest that the hydrophobic residues F108, F109, L112, and L116 in the α3 helix play a critical role in the structure of the PML monomer and, consequently, in the assembly of NBs.

### PML dimeric interface

**Fig. 3:**
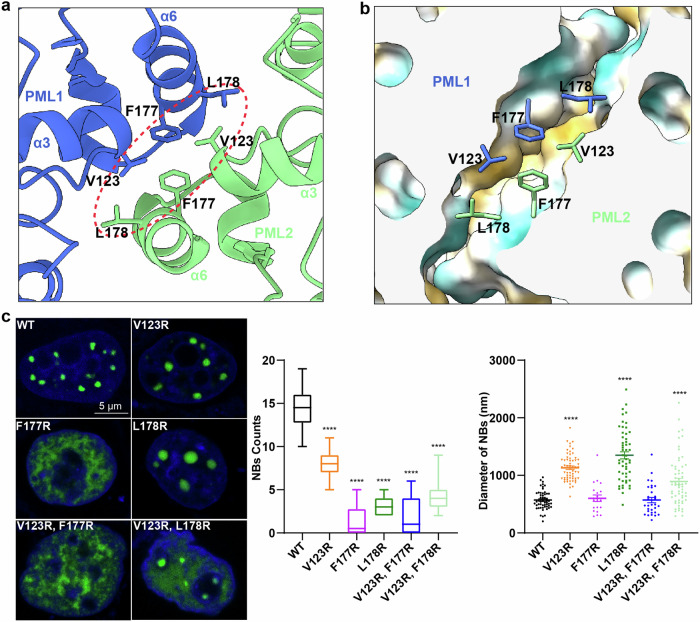
Examination of PML RBCC dimer interactions. **a** Detailed interactions in the hydrophobic interface of the PML dimer. **b** Slabbed view of PML1−PML2 interaction interface. The surfaces, defined by hydrophilic and hydrophobic residues, were colored in blue and yellow, respectively. **c** Fluorescence microscopy images of HeLa^*Pml−/−*^ cells expressing EGFP-PML, EGFP-PML_V123R_, EGFP-PML_F177R_, EGFP-PML_L178R_, EGFP-PML_V123R,F177R_ and EGFP-PML_V123R,L178R_. Scale bar, 5 µm. Statistical analysis of PML NB formation was conducted. All experiments were performed in independent replicates, with NB counts calculated from ≥ 15 nuclei. Data are means ± SEM. ****P < 0.0001 (derived from ordinary one-way ANOVA test). All experiments/results displayed in the main figure were conducted with PML isoform IV.

The current cryo-EM structure revealed that the PML monomer, which serves as the basic unit for assembling PML NBs, forms a functional PML dimer via two α6 helices (Fig. 3a, b). Specifically, V123, F177, and L178 within the α6 helix from one monomer interact with their counterparts from the other monomer, facilitating this dimerization (Fig. 3a, b). In order to characterize this further, we introduced single or double mutations on these residues. The central role of F177 in the hydrophobic core was confirmed as the F177R mutation completely inhibited NB formation (Fig. 3c). Similarly, point mutations V123R and L178R resulted in larger but fewer NBs (Fig. 3c). Supportively, double mutations of V123 and L178 exacerbated damage to NB formation, as evidenced by a significant reduction in NB numbers and increased PML diffusion in the background. These findings underscore the critical role of α6 helix and residues V123/F177/L178 in forming the PML dimeric interface.

### PML CC-based polymerization

**Fig. 4:**
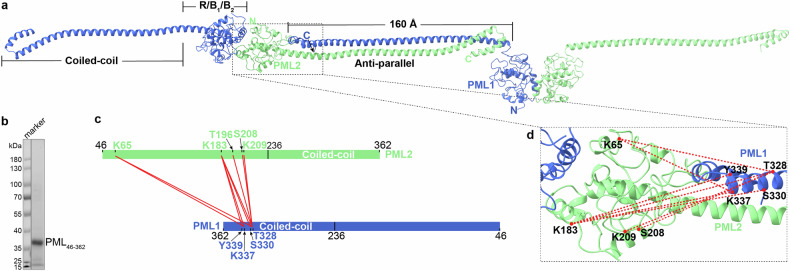
PML CC-driven polymerization. **a** Illustration of the CC-based PML polymerization. The dimeric interaction pattern of the CC domain was predicted by AlphaFold2, and the PML_R/B1/B2_ dimer represents the experimentally obtained cryo-EM structure. **b** SDS-PAGE analysis of the PML_46−362_ protein. **c** XL-MS analysis of the purified PML_46−362_ protein, with the identified inter-subunit XLs shown as red lines. The best E-value (1.00 × 10^−2^) and spec count of at least 2 were used as the threshold to remove extra XL-MS data with lower confidence. **d** Enlarged view of the anti-parallel PML1_46−360_−PML2_46−360_ interaction, with the detected XLs mapped on the model.

PML NBs are MLOs with diameters ranging from 0.1 µm to 2 µm. However, a PML monomer is only ~70 kDa. The molecular mechanisms of how a 70 kDa PML molecule could form an initial scaffold structure, and how these molecules could rapidly assemble into an MLO, are yet to be defined. What is the interaction pattern of the PML CC domain, and how does it contribute to overall NB formation? Surprisingly, despite the strong CC–CC interaction^75–78^ (and more figures below), the density for the PML_222–256_ was not visible in our cryo-EM map, where residues 236–256 are part of the CC domain. Furthermore, based on the current model, the C-terminal S221 residues from the two monomers locate on opposite sides of the PML dimer, facing away from the dimeric interface. This suggests that the two PML CC helices following S221 might be positioned on opposite sides of the PML dimer, resembling the arms of an octopus. (Fig. 1f). In order to visualize how these long and flexible PML CC helices might interact with each other, we used AlphaFold2 multimer module to predict the interaction between PML CC domains (Fig. 4a). Interestingly, an anti-parallel CC dimer was proposed based on the AlphaFold2 prediction, aligning with our cryo-EM findings (Fig. 4a).

In order to validate the anti-parallel CC–CC interaction, we purified the truncated protein PML_46–362_ (Fig. 4b), which encompasses the complete RBCC domain. This protein was then subjected to XL-MS analysis using crosslinker BS3 (11.4 Å) (Fig. 4c; Supplementary Table S5). The CC domain is ~160 Å in length (Fig. 4a). Notably, the simulated PML_46–360_ model shows a discrepancy compared to the actual structure, resulting in slightly greater Cα–Cα distances (40–60 Å) between the interacting subunits. The results revealed intensive interactions between the N-terminal residues situated in the RING (K65), α6 (K183), and B2 (T196/S208/K209) regions with those in the C-terminal CC region (T328/S330/K337/Y339) (Fig. 4d), further supporting the anti-parallel dimerization of the PML CC domains.

### Functional characterization of PML CC domain

**Fig. 5:**
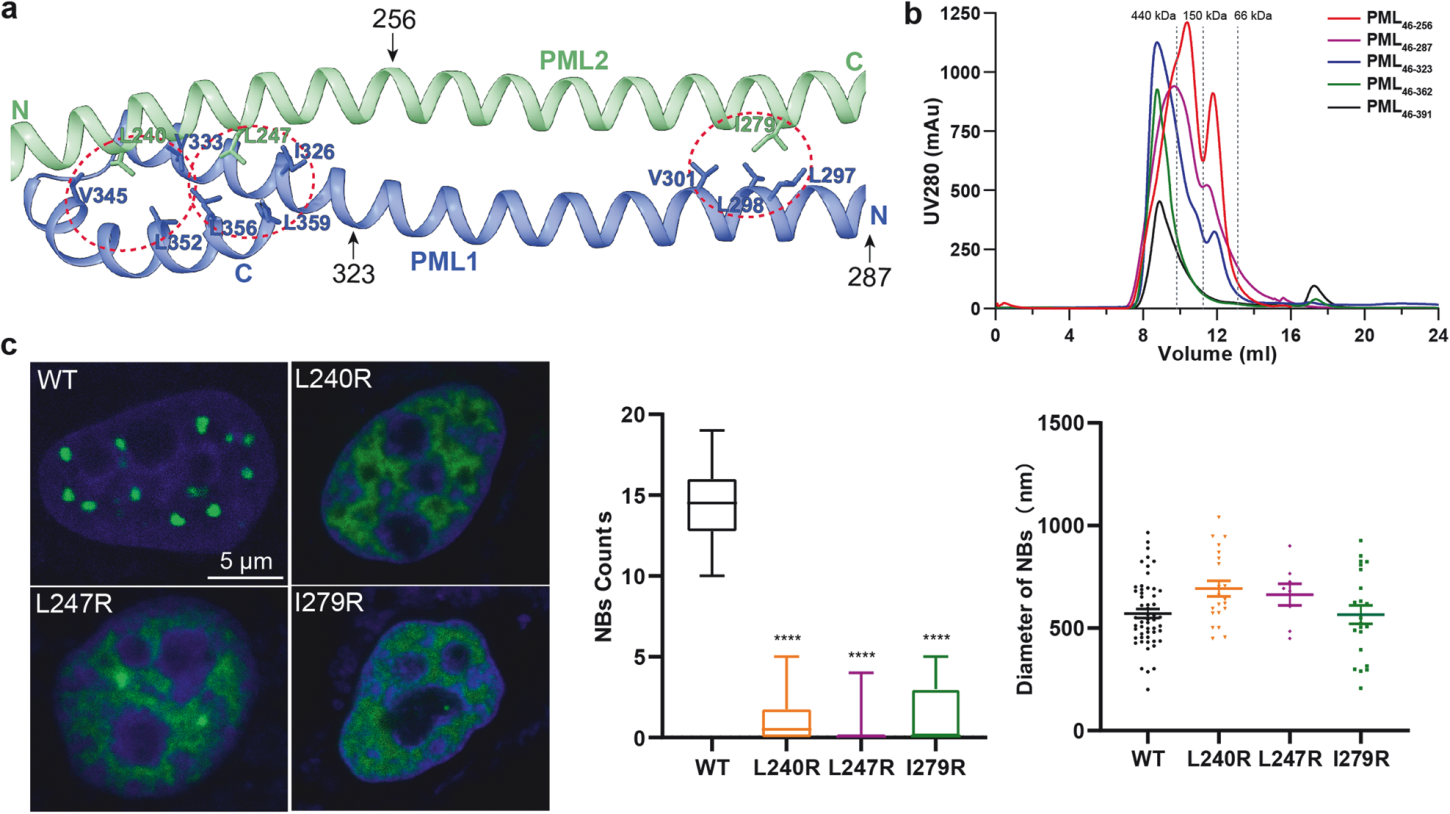
Functional characterization of PML CC domain. **a** Three hydrophobic pockets were observed in PML CC dimer. PML1 and PML2 were shown in cartoon representation and colored in blue and green, respectively. Hydrophobic residues in the hydrophobic pockets were annotated and shown in stick representation. **b** Size-exclusion analytical chromatography of the purified PML_46−256_, PML_46−287_, PML_46−323_, PML_46−362_, and PML_46−391_. The calibrated molecular weights of each elution/fraction were shown above the chromatographic traces. The N-termini of the aforementioned truncation mutants were all fused with an MBP tag. **c** Fluorescence visualization of HeLa^*Pml−/−*^ cells expressing EGFP-PML, EGFP-PML_L240R_, EGFP-PML_L247R_, and EGFP-PML_I279R_. Scale bar, 5 µm. Statistical analysis of PML NB formation was conducted. All experiments were performed in independent replicates, with NB counts calculated from ≥ 15 nuclei. Data are means ± SEM. ****P < 0.0001 (derived from ordinary one-way ANOVA test). All experiments/results displayed in the main figure were conducted with PML isoform IV.

To explore the function of PML CC domain (Fig. 5a), we generated truncated mutants at varying lengths: PML_46–256_, PML_46–287_, PML_46–323_, PML_46–362_, and PML_46–391_ (Fig. 5b). We expressed and purified these truncation mutants, demonstrating that the length of the CC domain is positively correlated with the aggregation state of the PML. The CC domain may significantly influence the recruitment of PML molecules, thereby affecting its oligomerization (Fig. 5b). We further analyzed the structure of PML CC dimer predicted by AlphaFold2 and observed three important hydrophobic pockets in between PML CC domains (Fig. 5a; Supplementary Fig. S4): (i) residue L240 of PML2 formed a hydrophobic pocket with residues V345, V333, and L352 of PML1; (ii) residue L247 of PML2 interacted with residues I326, L356, and L359 of PML1; (iii) residue I279 of PML2 formed a hydrophobic pocket with residues L297, L298, and V301 of PML1. Based on this observation, L240, L247, and I279 were mutated to the hydrophilic amino acid Arg. Their impacts on NB formation were monitored in *Pml* knockout HeLa (HeLa^*Pml−/−*^) cells (Fig. 5c). Supportively, L240R, L247R, and I279R mutations consistently abolished the formation of NBs (Fig. 5c), indicating the crucial importance of these three hydrophobic pockets in CC polymerization in an anti-parallel manner.

**Fig. 6:**
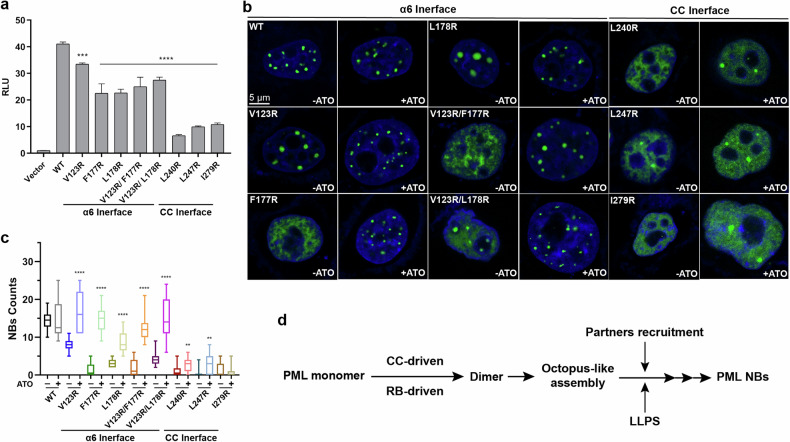
A working hypothesis for PML NB assembly. **a** Results of mammalian two-hybrid assay. Relative luciferase activities (RLUs) were used to evaluate the self-interaction of PML and its variant proteins. All data were normalized against the “Vector” group (pACT vector:pBIND-PML interaction = 1), with PML-WT serving as a positive control. All experiments have been conducted in at least three independent replicates. Data are means ± SEM. ***P < 0.001, ****P < 0.0001 (derived from ordinary one-way ANOVA test) were used to show statistical significance. **b**, **c** NB formation rescued by ATO. HeLa^*Pml−/−*^ cells expressing PML-WT and PML variants were exposed to 2 μM ATO for 1 h before visualization. Scale bar, 5 µm. Statistical analysis of PML NB formation was conducted. Data are means ± SEM. **P < 0.01, ****P < 0.0001 (derived from ordinary one-way ANOVA test). All experiments were performed in independent replicates, with NB counts calculated from ≥ 15 nuclei. All experiments/results displayed in the main figure were conducted with PML isoform IV. **d** The assembly process of PML NBs was summarized below. Formation of PML monomer: the RING, B1, and B2 domains orderly fold around the PML α3 and distribute on the outer surface of PML molecules. PML dimerization: the resulting PML monomers engage with each other via RB (RING, B1, B2, PML α6)- or CC-interaction interfaces to form a stable dimer. Octopus-like assembly: the CC domain of PML is flexible, and it further recruits and binds PML proteins in an anti-parallel orientation. Of note, although current results favored anti-parallel configuration, parallel CC–CC interaction could not be excluded and needs further characterization in the future. Partner recruitment: SUMO-conjugated PML recruits SIM-containing partners through non-covalent SUMO/SIM interactions. LLPS: by spatial multimerization, millions of PML molecules might polymerize into a higher-order complex, initiating LLPS and ultimately leading to the formation of PML NBs.

To investigate the differences between RB (RING and B-box1/2)- and CC-mediated dimerization, we performed a mammalian two-hybrid assay using full-length PML protein to determine how perturbations in the RB- and CC-dimeric interfaces impair PML–PML engagement and assembly. We expressed the wild-type PML (PML-WT), PML mutants in the RB region (i.e., V123R, F177R, L178R, V123R/F177R, and V123R/L178R), as well as PML mutants in the CC domain (i.e., L240R, L247R, and I279R) in HeLa^*Pml−/−*^ cells. As shown in Fig. 6a, the CC mutations L240R, L247R and I279R significantly impaired PML self-interaction (i.e., 83.88%, 75.76%, and 73.65% reduction, respectively, compared to control). In comparison, RB mutants caused relatively minor disruption in PML dimerization (18.5%, 45.15%, 44.90%, 39.08%, and 33.11% reduction, respectively). This suggested that the CC-mediated dimerization might be more important than that mediated by RB (Fig. 6a). To corroborate this, we carried out an As_2_O_3_-faciliated PML NB reappearance experiment (Fig. 6b, c), wherein arsenic trioxide (ATO) was used to facilitate NB formation by crippled PML due to RB or CC mutations. The more easily the PML NBs reassemble, the less severe the disruption caused by the RB or CC mutation. In this way, the ATO-rescue results could help differentiate which dimeric interface was more critical. Consistent with the mammalian two-hybrid assay, it was clear that the NBs formed by PML with RB mutations could be swiftly rescued by ATO (Fig. 6b, c). In marked contrast, the mutants in CC displayed the opposite effect (Fig. 6b, c), reinforcing the importance of the CC-mediated octopus-like assembly in PML NB formation (Fig. 6d).

## Discussion

The PML NB is an MLO that integrates numerous regulatory functions within the cell nucleus. This supramolecular assembly complex forms a robust active network within cells, regulating numerous signaling pathways. Therefore, PML NB serves as an important target in the treatment of leukemia and solid tumors^79–87^. In this study, we present the first cryo-EM structure of the PML RBCC, offering new insights into the structure and functions of PML CC domains and non-conserved sequences within the RBCC. In previous studies, the PML RING, B1 and B2 domains were individually determined by X-ray crystallography^60,61,68^. These crystal structures have led to a consensus that PML RING, B1, and B2 can mediate homotypic PML–PML interactions. However, due to the limited size of the RBCC fragment, the potential interplay between these subdomains, which could culminate in the formation of a macromolecular complex of up to 2 μm, remains unclear.

Our cryo-EM structure showed that in the monomer of PML RB, the RING, B1, and B2 domains are organized around the α3 helix and are situated on the outer surface of the PML molecule. This arrangement aligns with their physiological functions, enabling the RING domain to bind UBC9 for PML SUMOylation and the B2 domain to be targeted by ATO^68^. Furthermore, the published RING and B1 interfaces are solvent-exposed, providing ample space for further oligomerization. Subsequently, PML dimers are formed through hydrophobic interactions centered on the α6 helix interface. Structure-based perturbation of the α3 and α6 helices causes severe damage to NB formation, reiterating the importance of these secondary structural elements. Circular dichroism (CD) spectroscopy confirmed that the structure-based Ala and Arg substitutions had minimal impact on the expression, purification, or overall folding of the PML protein (Supplementary Fig. S5). Interestingly, the current structure revealed that the CC domain of PML is more flexible than previously assumed. In the context of the PML dimer, the CC helices extend outward like two long tails from both sides, away from the α6–α6 dimeric interface. This unique spatial arrangement may play a crucial role in recruiting neighboring PML molecules/partners through remarkable anti-parallel interactions mediated by the enriched Leu/Val residues.

In 2018/2019, the crystal structures of the PML-RING and PML-B1 domains were successively determined, revealing unique oligomerization patterns for both^60,61^. The PML-RING tetramer plays a pivotal role in PML NB assembly, with only aggregated PML undergoing SUMOylation. This process recruits proteins with SIM and other partners to assemble into mature PML NBs. Moreover, it was found that the oligomerization of the B1 domain is also crucial for PML’s function. These perspectives align with our findings, and the assembly mechanism of PML NBs has been further refined. The PML dimers mediated by the α6 helix might be drawn closer together through the robust and persistent recruitment of the CC domain, akin to the way an octopus pulls objects to itself. In turn, the binding interface between the RING and B1 could facilitate further PML transition from oligomerization to higher-order assembly. This process further completes the assembly and stacking of PML proteins. Simultaneously, the recruitment of partner proteins through partner-SIM and PML-SUMO interactions^88,89^, along with the effect of LLPS, promotes PML NB formation, with diameters ranging from 0.1 µm to 2 µm. In order to check this, the partners SP100 and SUMO2 were co-expressed with PML-WT and PML variants L116E and L178R. Interestingly, we observed that the granules of PML-WT enlarged in the presence of SP100 and SUMO2 proteins (Supplementary Fig. S6a–c). In contrast, when PML oligomerization was disrupted, the sizes of crippled PML NBs remained unchanged regardless of the presence of partner proteins (Supplementary Fig. S6a, b). This reiterates that partner recruitment might be an auxiliary step following octopus-like oligomerization (Fig. 6d). In 2023, the crystal structure of the monomeric PML-B2 was also resolved^68^. The research team employed AI methods to simulate the binding mode between ATO and PML-B2, suggesting that trivalent arsenic ions bind directly to the PML trimer centered on Cys213, thereby exerting a targeted therapeutic effect^68^. However, it is worth pointing out that the proposed binding mode between PML and ATO remains at the molecular simulation stage. Research on the mechanism of ATO targeted therapy for APL requires further exploration of the interaction between PML-RARα and ATO directly to finally unravel its mystery.

Significantly, our exploration of the functional relationships among the various domains of the PML protein is constrained due to limitations in structural resolution of this relatively small complex (less than 50 kDa for the PML dimer). Furthermore, there are no reports on the structure of complexes formed by small-molecule drugs with the PML-RARα protein, indicating a substantial gap in our understanding of the therapeutic mechanisms of APL. Despite extensive research into the structure and function of PML, over the past three decades, significant challenges have persisted in the expression and purification of large segments and the full-length protein of PML due to its unique aggregation property. This has imposed considerable limitations on our exploration of its structure and function, leaving a significant gap in our understanding of the molecular mechanisms underlying the assembly of PML NBs. The PML_46–256_ dimer, however, provides the first revelation of the 3D structure of the PML protein at the molecular level. It elucidates the folding and distribution of the RING, B1, B2, and CC domains, and also unprecedentedly refines the understanding of PML NB assembly by proposing a unique octopus-like assembly mechanism. This can provide a solid theoretical foundation for targeted therapies and prognosis of AML focused on PML NBs, as well as other PML-related solid tumors.

## Materials and methods

### Expression and complex purification

The human *PML*_*46–256*_, *PML*_*46–287*_, *PML*_*46–323*_, *PML*_*46–362*_ and *PML*_*46–391*_ genes were cloned into the pRSFDuet-1 vector with an N-terminal MBP tag, and a C-terminal 6× His tag (ClonExpress One Step Cloning Kit, Vazyme). These constructs were individually transformed into *Escherichia coli* BL21(DE3) cells (Sangon) for protein production. Protein expression was induced with 100 µM isopropyl β-d-1-thiogalactopyranoside (IPTG, Sangon) when the OD_600_ reached 0.7. The culture was grown at 16 °C for 16 h, then harvested by centrifugation at 4000 rpm for 30 min and stored at –80 °C.

The cell pellets were lysed in 20 mM Tris-HCl, pH 7.4, 100 mM NaCl, 20 mM ZnCl_2_, 10% glycerol supplemented with 1 mM phenylmethylsulfonyl fluoride. The bacterial cells were lysed with cell cracker (JNBIO) applying 20 kg/cm^2^ pressure at 4 °C, followed by centrifugation at 35,000× *g* for 40 min. Supernatant was subsequently loaded onto a pre-equilibrated amylose column (GE Healthcare) at 4 °C. The resin was washed with ~20 column volumes of 20 mM Tris, pH 7.4, 500 mM NaCl, 20 mM ZnCl_2_, 10% glycerol, and then the protein was eluted with 5 column volumes of lysis buffer containing 10 mM maltose. At this step, the proteins with MBP tag were purified. The MBP tag was removed by the thrombin enzyme at 4 °C overnight, and then the cleaved proteins were collected using Ni-NTA beads (Smart-Lifesciences) and subsequently eluted with lysis buffer containing 250 mM imidazole. The proteins were concentrated and subjected to a Superdex^TM^ 200 Increase 10/300 GL column (GE Healthcare) in 20 mM HEPES, pH 7.4, 100 mM NaCl, 4% glycerol. The purified MBP-PML_46–256_ dimer fractions were collected and concentrated for cryo-EM analysis. The purified MBP-PML_46–256_ and PML_46–256_ monomers, MBP-PML_46–256_ and PML_46–256_ dimers, and PML_46–362_ protein fractions were collected and then concentrated for XL-MS assays.

### XL-MS analysis

The MBP-PML_46–256_ and PML_46–256_ monomers, MBP-PML_46–256_ and PML_46–256_ dimers, and PML_46–362_ proteins were crosslinked by reacting with BS3 (Sigma-Aldrich) at a final concentration of 2 mM (4 °C, 2 h), respectively. These reactions were terminated with 50 mM Tris-HCl (pH 7.5) at 4 °C for 1 h. The crosslinked proteins were first precipitated using acetone, followed by drying and subsequent dissolution in a solution containing 8 M urea and 100 mM Tris-HCl (pH 8.5). Reduction and alkylation were performed by adding 5 mM TCEP (Thermo Fisher Scientific) and 10 mM Iodoacetamide (Sigma), respectively, followed by incubation at room temperature for 30 min. The protein mixture was then diluted by 4-fold and subjected to overnight digestion with Trypsin at a ratio of 1:50 (w/w) (Promega). The resulting digested peptide solutions were purified using a MonoSpin^TM^ C18 column (GL Science, Tokyo, Japan) and subsequently dried. Analysis of the peptide mixture was carried out using a custom-made analytical column measuring 30 cm in length (packed with ReproSil-Pur C18-AQ 1.9 µm resin from Dr. Maisch GmbH, 75 µm ID) coupled with an Easy-nLC 1200 nano HPLC system (Thermo Fisher Scientific, San Jose, CA, USA) for MS analysis.

LC/tandem MS (MS/MS) analysis was performed by an Easy-nLC 1200 nano HPLC (Thermo Fisher Scientific, San Jose, CA, USA). The analytical column temperature was set at 55 °C during the experiments. Data-dependent MS/MS analysis was performed with a Q Exactive Orbitrap mass spectrometer (Thermo Fisher Scientific, San Jose, CA, USA). Peptides eluted from the LC column were directly electrosprayed into the mass spectrometer with the application of a distal 2.2-kV spray voltage. A cycle of one full-scan MS spectrum (m/z 300–1800) was acquired followed by top 20 MS/MS events, sequentially generated on the first to the twentieth most intense ions selected from the full MS spectrum at a 28% normalized collision energy. Full scan resolution was set to 70,000 with automated gain control target of 3e6. MS scan functions and LC solvent gradients were controlled by the Xcalibur data system (Thermo Fisher Scientific). Data analysis was performed as follows. Peptides containing the isopeptide bonds were identified using pLink2 software (pFind Team, Beijing, China) as described previously^90,91^. Carbamidomethylation of cysteine and oxidation of methionine were set as variable modifications. The results were filtered by applying a 5% FDR cutoff at the spectral level.

### PML NB biogenesis and fluorescence image acquisition

The WT PML-IV (UniProt code, P29590-5) and its mutants were constructed into the pEGFP-C1 vector containing an N-terminal EGFP tag, and then transfected into HeLa^*Pml−/−*^ cells for expression. Concerning ATO response, the cells harboring PML-WT and its variants were incubated with 2 μM As_2_O_3_ for 1 h before visualization. For the co-localization assay, the PML partner proteins SUMO2 and SP100 with mCherry tag were constructed into pFLAG-CMV4 vectors individually. The mCherry-SUMO2 and SP100-mCherry were co-transfected into the HeLa^*Pml−/−*^ cells with GFP-PML-WT or their mutants at 1:1 molar ratio. The endogenous *PML* gene in the HeLa^*Pml−/−*^ cells was knocked out using CRISPR-Cas9 strategy^4,60,92^. The cells were grown in Dulbecco’s modified Eagle’s medium (DMEM) supplemented with 10% fetal bovine serum. After 24 h of cell expression, cells were fixed with 4% paraformaldehyde. Nuclei were stained with 4’,6-diamidino-2-phenylindole (DAPI), and finally subjected to fluorescence examination. The slides were imaged using either a Leica TCS SP8 or a Zeiss LSM870.

### CD spectroscopy

The WT PML_46–256_ and PML_46–256_ variants were prepared by desalting the bacterially expressed recombinant proteins and diluting them to 0.1 mg/mL using a buffer containing 20 mM Tris (pH 7.5) and 20 mM NaCl. Far UV CD spectra were recorded from 185 nm to 260 nm at 20 °C with a time constant of 1 s using a Chirascan spectrometer (Applied Photophysics Ltd). The spectra were averaged from no fewer than three scans and presented as mean residue molar ellipticity (θ) (deg.cm^2^ dmol^−1^). Secondary structure content was estimated using the CD deconvolution program CDNN.

### Mammalian two-hybrid assay

This assay was performed using the CheckMate^TM^ Mammalian Two-Hybrid System (Promega) in HeLa^*Pml–/–*^ cells. The cDNA of full-length PML-WT and mutants were inserted into pBIND and pACT vectors, respectively. HeLa^*Pml–/–*^ cells were then transfected with plasmids pG5-luc, pBIND-PML_WT_/PML_mutant_ and pACT-PML_WT_/PML_mutant_ (1:1:1) using the liposome lipo3000 transfection method (Life Technology). The HeLa^*Pml–/–*^ cells were harvested after 24 h transfection, and luciferase activities were monitored using the Dual-Luciferase Reporter Assay System (Promega).

### Cryo-EM sample preparation and data collection

To prepare the cryo-EM sample of MBP-PML_46–256_, freshly purified samples at a concentration of ~3 mg/mL were applied on a plasma-cleaned holy carbon grid (R2/1, Au, 300 mesh; Quantifoil). The grid was blotted using a Vitrobot Mark IV (Thermo Fisher Scientific) for 2 s at 100% humidity, and then plunge frozen into liquid ethane. Cryo-EM movies of the samples were collected on a Titan Krios electron microscope (Thermo Fisher Scientific) operated at an accelerating voltage of 300 kV. The movies were collected at a magnification of 81,000× and recorded on a K3 direct electron detector (Gatan) operated in the counting mode (yielding a pixel size of 0.89 Å), and under a low-dose condition in an automatic manner using EPU 2.11 software (Thermo Fisher Scientific). Each frame was exposed for 0.08 s, and the total exposure time was 2.41 s, leading to a total accumulated dose of 54 e^–^/Å^2^ on the specimen. For tilt movies, data were collected with stage tilt at 40° while other conditions remained unchanged.

### Cryo-EM image processing

We collected 2439 non-tilt movies and 2360 40°-tilt movies. Motion correction was performed using MotionCor2^93^ embedded in Relion 4.0^94^, and CTF estimation was carried out using CTFFind 4.1.8^95^. For tilt particles, we used goCTF 1.1.0^96^ for CTF estimation. Utilizing cryoSPARC v4^97^, we picked ~2.2 million particles from both datasets, and after 2 rounds of reference-free 2D classification, about 1 million good particles remained. We then generated initial models using ab-initio reconstruction in cryoSPARC v4 for subsequent 3D reconstruction. All the refinements were performed in Relion 4.0 unless stated otherwise. The good particles were re-extracted, re-centered, and subjected to two rounds of 3D classification to remove incomplete particles, leaving 307,216 particles. Given the clear 2-fold symmetric features of the protein revealed by the 2D class averages, we performed 3D refinement with imposed C2 symmetry. After local refinement, two rounds of CTF refinement and polishing, we obtained a cryo-EM map of PML_46–256_ in dimer configuration from 307,216 particles at a nominal resolution of 5.3 Å. The overall resolution of the cryo-EM map was determined based on the gold-standard criterion using a Fourier shell correlation of 0.143. This map was further sharpened and enhanced using DeepEMhaner 0.14^98^.

### Atomic model building

To build an atomic model for the PML_46–256_ complex, we initially utilized the model predicted by AlphaFold2^66,67^, which coordinates the structural information from the available X-ray structures for RING, B1, and B2 domains^60,61,68^. This model was then docked into the density map via rigid body fitting using UCSF Chimera 1.15^99^, and any residues outside the density were trimmed. Subsequently, we performed flexible fitting of the model against the map using Rosetta^100^. We then inspected and locally refined the model against the map in Coot 0.9.7^101^ and ISOLDE^102^. For the final stage of complete model refinement, we employed the Real_space_refine Module^103^ in Phenix 1.19.2-4158^104^, with the constraint of the map. The structural figures were rendered using PyMOL (https://pymol.org/2/) and ChimeraX^105^.

### Dimeric PML_46–360_ model prediction and building

The PML_46–221_ dimer was experimentally obtained by cryo-EM. The dimeric PML_222–360_ was extracted from the PML_46–360_ dimer, which was predicted at Shanghai Jiao Tong University’s Center for High-Performance Computing, using the multimer model in AlphaFold (v2.3.1)^67,106^. The multimer model in AlphaFold (v2.3.1) predicted five PML CC models, ranked from rank_001 to rank_005, with confidence decreasing from rank_001 to rank_005. All predictions indicated an anti-parallel CC–CC interaction between different PML CC molecules, and the rank_001 model of the PML_46–360_ dimer was chosen for the next step. First, we aligned the EM model of PML_46–221_ with the predicted structure of dimeric PML_46–360_, while also attempting to match the PML_46–360_ XL-MS interaction pattern. We then deleted amino acids 46–221 from the predicted PML_46–360_ structure. By combining the EM model of PML_46–221_ with the extracted PML_222–360_ model, we ultimately obtained the simulated dimeric PML_46–360_ model.

## Supplementary information

Supplementary Figures and Tables

## Data Availability

All data are available in the main text or supplementary materials. Materials are available from the corresponding authors upon reasonable request. The density map and structure coordinates have been deposited to the Electron Microscopy Data Bank (EMDB) and the Protein Data Bank (PDB) under accession numbers EMD-39571 and 8YTC, respectively.
