# Supplementary material for: Cryo-EM structure of PML RBCC dimer reveals CC-mediated octopus-like nuclear body assembly mechanism
Source: Cell Discov. 2024 Nov 25;10:118. doi: 10.1038/s41421-024-00735-3 (PMC11589706; doi:10.1038/s41421-024-00735-3)
Supplement: Supplementary file 1 — Supplementary Figures and Tables [file 41421_2024_735_MOESM1_ESM.pdf]

Figure S1 Tan et al.

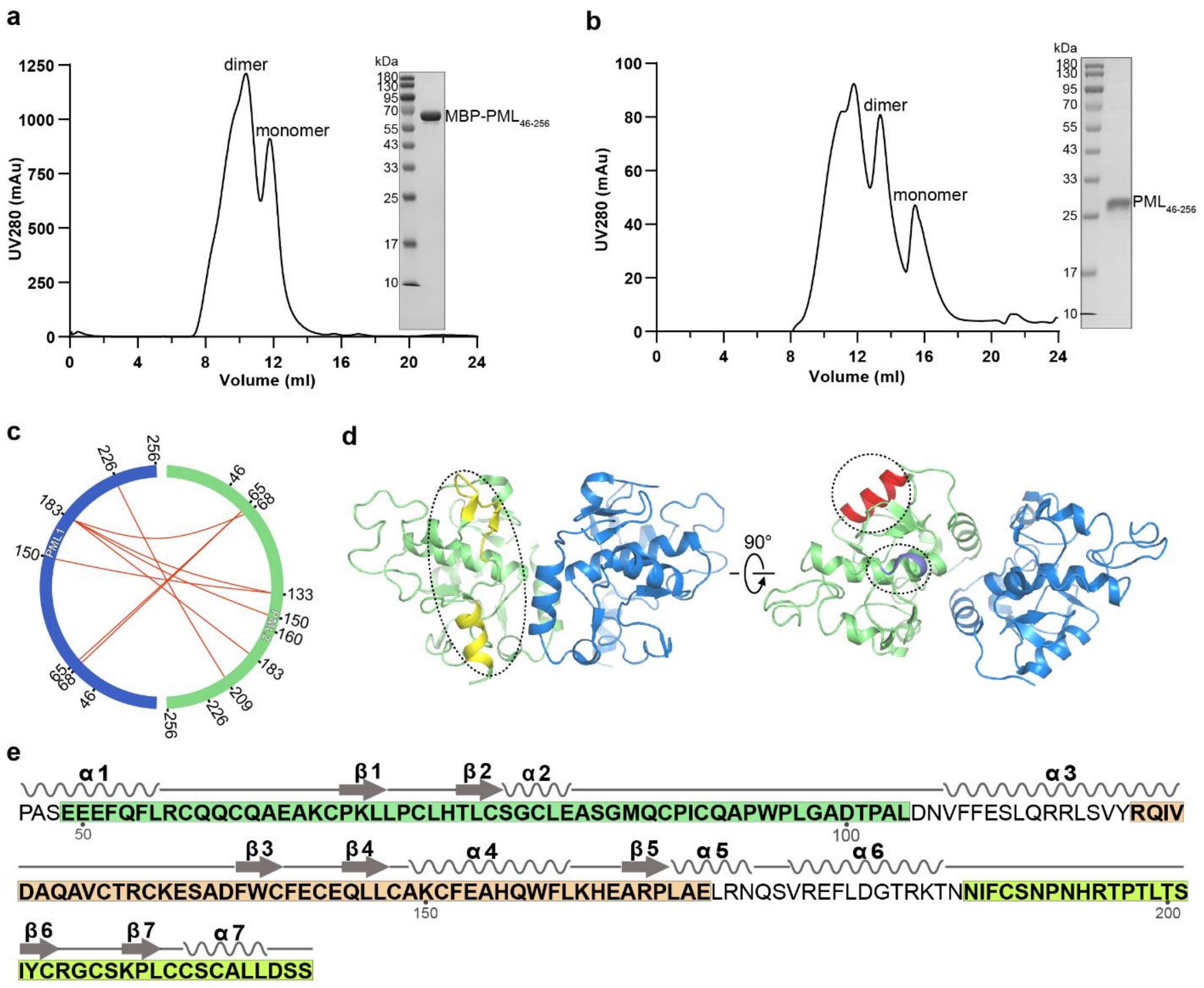

Fig. S1 Characterization and purification of the $PML_{46\text{-}256}$ dimer. a, Gel filtration characterization of the purified MBP-$PML_{46\text{-}256}$ protein on a Superdex 200 column and the accompanied SDS-PAGE analysis. **b,** Gel filtration and SDS-PAGE of $PML_{46\text{-}256}$. **c,** XL-MS analysis of the $PML_{46\text{-}256}$ dimer. The intermolecular cross-linked interactions were depicted as red lines. We used the best E-value (1.00×10^{-2}) and spec count of at least 2 as the threshold to remove XL-MS data with lower confidence. **d,** The published crystallographic RING (yellow)[1], B1 (red)[2] and B2 (purple)[3]interaction interfaces in the cryo-EM $PML_{46\text{-}256}$ model. **e,** PML amino acid sequence characterized by cryo-EM. The secondary structures were shown on top of the sequences, and the structure domains of RING (light green), B1 (tan) and B2

(yellow-green) are indicated, respectively.

Figure S2 Tan et al.

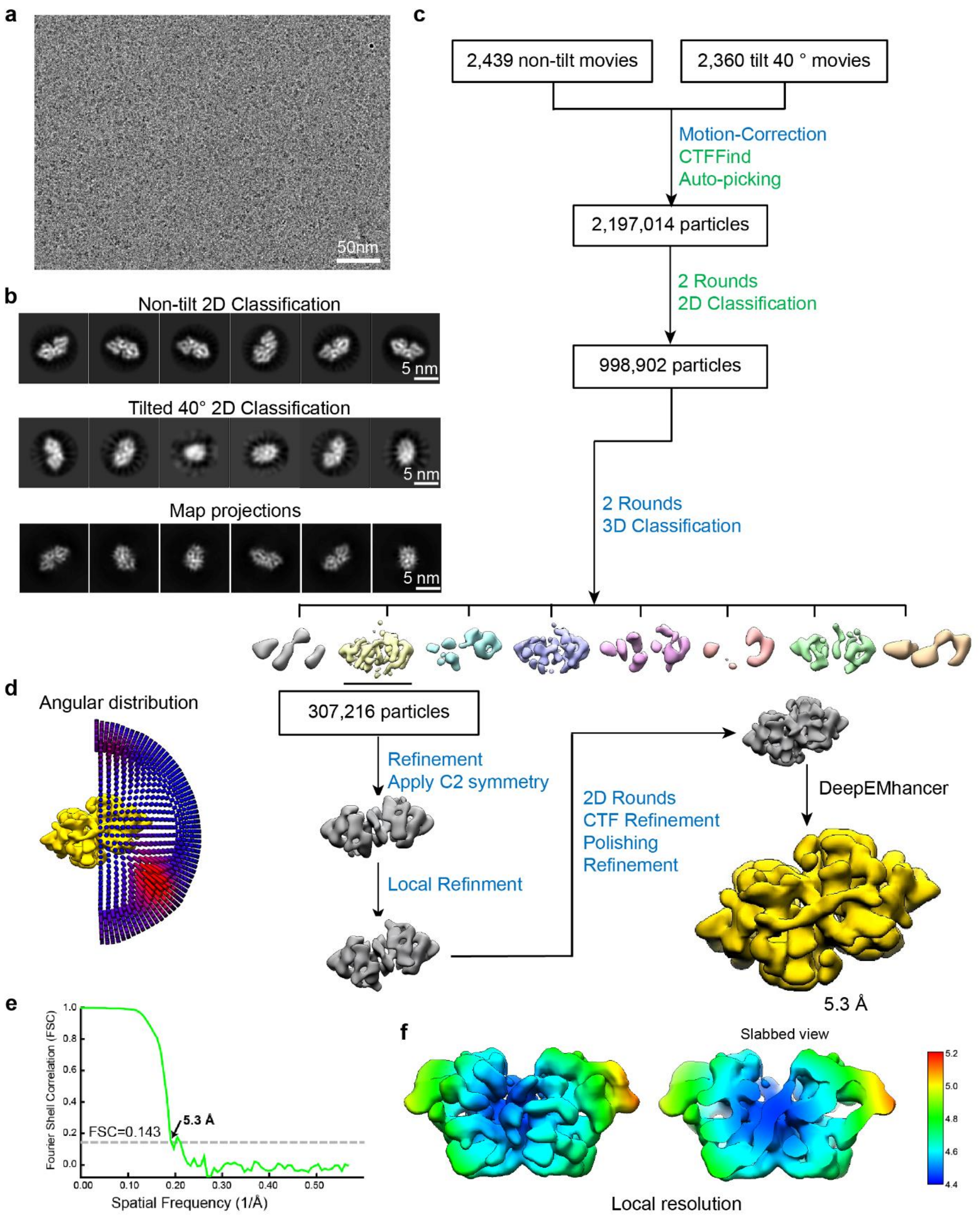

Fig. S2 Cryo-EM data processing procedure for PML$_{46\text{-}256}$ dimer. a, A representative cryo-EM micrograph of the PML$_{46\text{-}256}$ dimer, contrast enhanced with 5 Å lowpass filter on the original micrograph for better visualization. The scale bar represents 50 nm. **b,** Representative

projections of the final cryo-EM map of the PML_{46-256} dimer (bottom row) and the corresponding reference-free 2D class averages of non-tilt and tilt 40° dataset (top row) obtained from raw particles showing similar structural features. The scale bar represents 5 nm. **c,** Workflow of the cryo-EM data processing for the PML_{46-256} dimer, with processes handled by Relion depicted in blue, and those by cryoSPARC in green. **d,** Angular distribution of the PML_{46-256} dimer. **e,** Resolution estimation of the cryo-EM map of the PML_{46-256} dimer by the FSC at 0.143 criterion. **f,** Local resolution evaluation of the cryo-EM map of the PML_{46-256} dimer.

Figure S3 Tan et al.

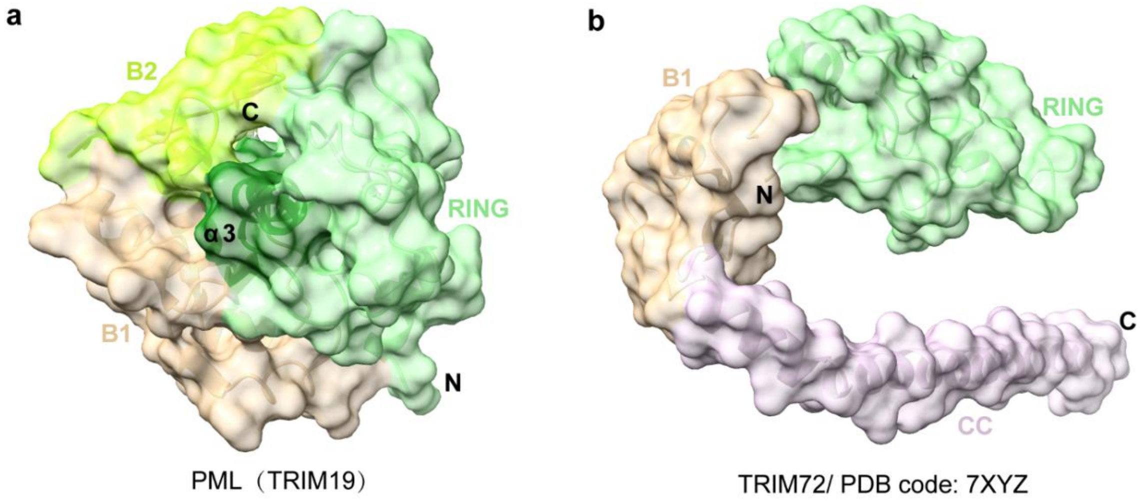

Fig. S3 The different structural characteristics of TRIM RBCC. a, Surface representation of the cryo-EM structure of $PML_{46\text{-}256}$ monomer, extracted from our dimer structure. **b,** Structure of the TRIM72 monomer, extracted from the TRIM72 tetramer X-ray structure (PDB ID: 7XYZ). The RING domain is shown in light green, B1 in tan, B2 in yellow-green, and CC in purple.

Figure S4 Tan et al.

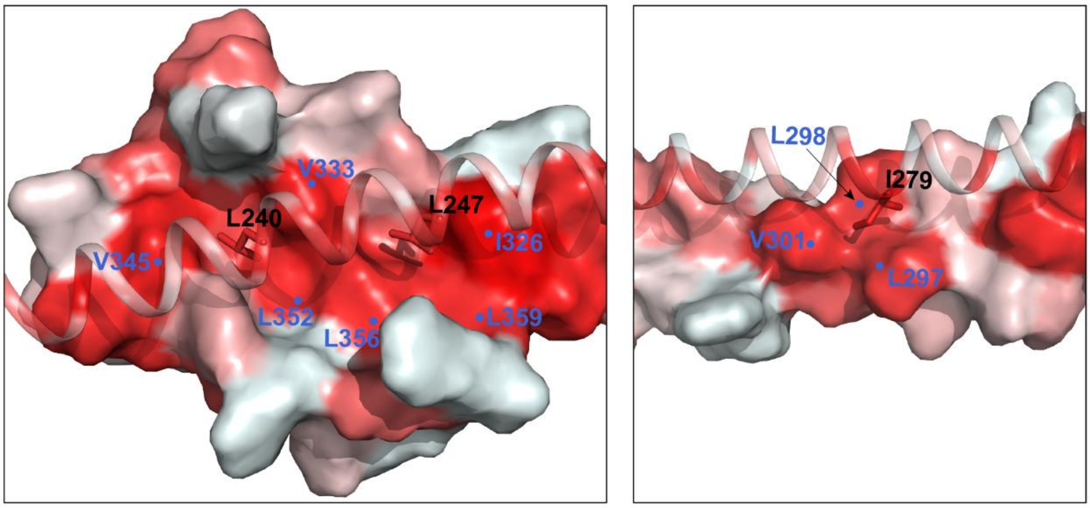

Fig. S4 Hydrophobic pockets of the PML CC dimer. Left panel, Pocket 1 and Pocket 2 were delineated by V333/V345/L352 and L240, and by I326/L356/L359 and L247, respectively. The PML1 and PML2 subunits were shown in surface representation and cartoon representations, respectively. Hydrophobicity was colored in red. Residues in PML1 and PML2 were annotated in blue and black, respectively. Right panel, Pocket 3 delineated by L297/L298/V301 and I279.

Figure S5 Tan et al.

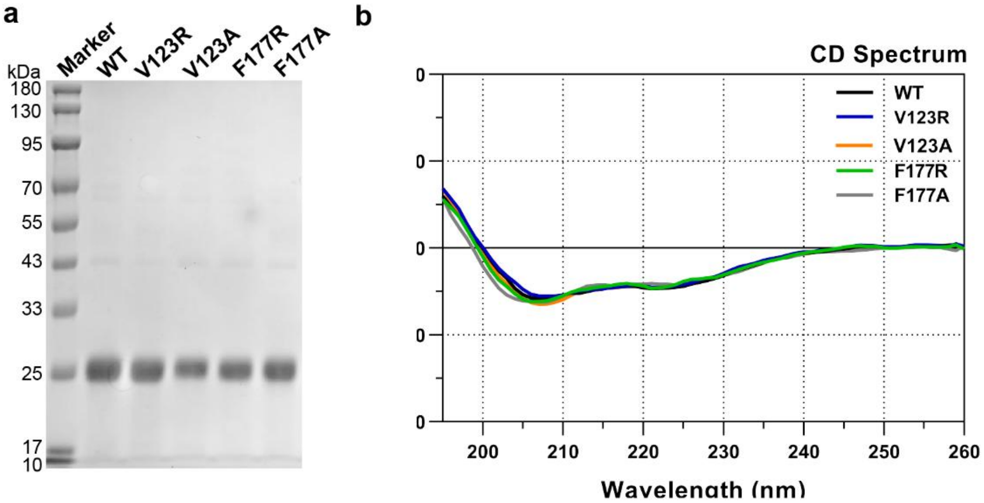

c

Estimate of protein secondary structure

	WT	V123R	V123A	F177R	F177A
**Wavelength**	195-260 nm	195-260 nm	195-260 nm	195-260 nm	195-260 nm
**Helix**	27.50%	27.90%	28.90%	27%	25.80%
**Antiparallel**	12.30%	13.00%	11.90%	12.60%	12.70%
**Parallel**	5.80%	6.00%	5.80%	5.70%	5.50%
**Beta-Turn**	20.60%	20.00%	21.00%	21.70%	22.70%
**Rndm. Coil**	32.80%	32.00%	31.60%	32.40%	32.60%
**Total Sum**	99.00%	99.00%	99.30%	99.30%	99.30%

Fig. S5 CD characterization of the PML-WT and variant proteins with Ala or Arg substitutions. a, SDS-PAGE analysis of purified WT PML_{46-256}, PML_{46-256}-V123R, PML_{46-256}-V123A, PML_{46-256}-F177R and PML_{46-256}-F177A proteins. **b,** CD characterization of WT PML_{46-256} and its variant proteins. **c,** The secondary structure content estimated by a circular dichroism deconvolution program CDNN.

Figure S6 Tan et al.

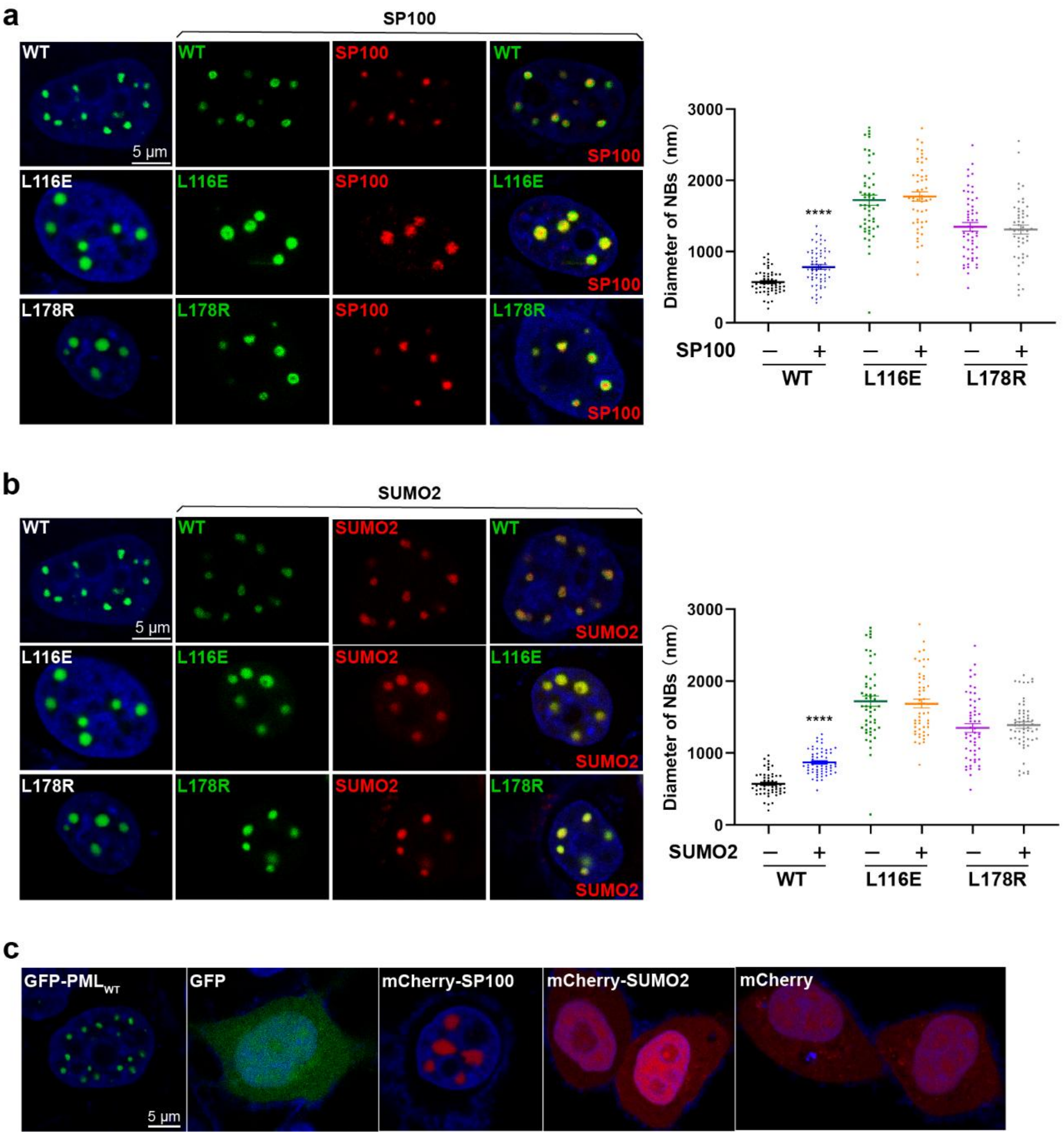

Fig. S6 Partner proteins and PML NB biogenesis. a, Colocalization of expressed EGFP-PML, EGFP-PML$_{L116E}$, or EGFP-PML$_{L178R}$ (green) and SP100-mCherry (red) in HeLa$^{Pml-/-}$ cells. Left panel, representative views by fluorescence microscopy. Right panel, Statistical analysis of NB size in the absence/presence of partner protein expression. **b,** Co-expression of PML (green) and mCherry-SUMO2 (red) in HeLa$^{Pml-/-}$ cells. Left panel, fluorescence visualization. Right panel, statistical analysis. DAPI is in blue. Scale bars are 5 μm. All experiments were performed in at least three independent replicates, with NB size

analysis calculated from ⩾15 nuclei. Values are presented as means ± S.E. ****, $p < 0.0001$. All experiments/results displayed in the main figure were conducted with PML isoform IV. **c,** Additional control experiments in fluorescence microscopy analysis: Vectors expressing EGFP-WT alone, EGFP, SP100-mCherry, mCherry-SUMO2, or mCherry proteins were transiently transfected into HeLa$^{Pml-/-}$ cells. Scale bars are 5 μm.

Table S1. Cryo-EM data collection, model refinement, and validation statistics

	PML_{46-256} Dimer (PDB: 8YTC)
**Data collection and processing**	
Magnification	81,000
Voltage (kV)	300
Electron exposure ($e^-/Å^2$)	54
Defocus range (μm)	-1 ~ -3
Pixel size (Å)	0.89
Symmetry imposed	C2
Initial particle projections (no.)	2,197,014
Final particle projections (no.)	307,216
Map resolution (Å)	5.3
FSC threshold	0.143
Map resolution range (Å)	4.4-5.2
**Refinement**	
Initial model used	AlphaFold (P29590)
Model resolution (Å)	5.3
FSC threshold	0.5
Map sharpening *B* factor ($Å^2$)	-397
Model composition	
Non-hydrogen atoms	2,604
Protein residues	332
Lipid	0
Water	0
B factors ($Å^2$)	
Protein	173.67
Ligand	0
R.m.s. deviations	
Bond lengths (Å)	0.003
Bond angles (°)	0.581
Validation	
MolProbity score	1.66
Clashscore	5.85
Rotamer outliers (%)	0.68
Ramachandran plot	
Favored (%)	95.06
Allowed (%)	4.94
Disallowed (%)	0.00

Table S2. XL-MS detected intra-subunit interactions of MBP-PML$_{46-256}$ monomer*

Protein1(site)-protein2(site)	Peptides	Best E-value	Spec count	Cα-Cα distance (Å)
PML(183)- PML(209)	KTNNIFCSNPNHR(1)-GCSKPLCCSCALLDSSHSELK(4)	1.49E-24	46	15.8
PML(65)- PML(160)	CQQCQAEAKCPK(9)-CFEAHQWFLKHEAR(10)	3.01E-11	5	26.5
PML(133)- PML(209)	CKESADFWCFECEQLLCAK(2)-GCSKPLCCSCALLDSSHSELK(4)	8.30E-27	5	31.2
PML(183)- PML(226)	KTNNIFCSNPNHR(1)-PLCCSCALLDSSHSELKCDISAEIQQR(17)	2.14E-19	16	-
PML(65)- PML(183)	CQQCQAEAKCPK(9)-KTNNIFCSNPNHR(1)	8.97E-18	38	25.7
PML(160)- PML(209)	CFEAHQWFLKHEAR(10)-GCSKPLCCSCALLDSSHSELK(4)	3.20E-27	14	11.4
PML(65)- PML(226)	CQQCQAEAKCPK(9)-PLCCSCALLDSSHSELKCDISAEIQQR(17)	1.60E-05	3	-
PML(65)- PML(209)	CQQCQAEAKCPK(9)-GCSKPLCCSCALLDSSHSELK(4)	2.38E-07	10	26.0
PML(65)- PML(133)	CQQCQAEAKCPK(9)-CKESADFWCFECEQLLCAK(2)	3.29E-08	3	23.1
PML(160)- PML(183)	CFEAHQWFLKHEAR(10)-KTNNIFCSNPNHR(1)	6.51E-29	51	8.7

*We used best E-value (1.00E-2) and Spec count of at least 2 as the thresholds to remove extra XL-MS data with lower confidence, and a Cα-Cα distance of less than 35 Å as the criterion to validate our cryo-EM model.

Table S3. XL-MS detected inter-subunit interactions of MBP-PML$_{46\text{-}256}$ dimer*

Protein1(site)-protein2(site)	Peptides	Best E-value	Spec count	Cα-Cα distance (Å)
PML1(133)-PML2(160)	CKESADFWCFECEQLLCAK(2)-CFEAHQWFLKHEAR(10)	5.65E-17	9	35.4
PML1(65)-PML2(68)	CQQCQAEAKCPK(9)-CPKLLPCLHTLCSGCLEASGMQCPICQAPWPLGADTPALDNVFFESLQR(3)	1.35E-08	13	28.9
PML1(160)-PML2(226)	CFEAHQWFLKHEAR(10)-GCSKPLCCSCALLDSSHSELKCDISAEIQQR(21)	3.35E-09	3	-
PML1(150)-PML2(183)	ESADFWCFECEQLLCAKCFEAHQWFLK(17)-KTNNIFCSNPNHR(1)	1.64E-13	4	33.9
PML1(209)-PML2(226)	GCSKPLCCSCALLDSSHSELK(4)-GCSKPLCCSCALLDSSHSELKCDISAEIQQR(21)	2.22E-10	6	-
PML1(183)-PML2(183)	KTNNIFCSNPNHR(1)-KTNNIFCSNPNHR(1)	9.36E-13	66	22.5
PML1(160)-PML2(160)	CFEAHQWFLKHEAR(10)-CFEAHQWFLKHEAR(10)	5.31E-04	7	33.3
PML1(209)-PML2(209)	GCSKPLCCSCALLDSSHSELK(4)-GCSKPLCCSCALLDSSHSELK(4)	2.75E-10	11	36.3
PML1(133)-PML2(183)	CKESADFWCFECEQLLCAK(2)-KTNNIFCSNPNHR(1)	1.12E-31	13	27.9
PML1(68)-PML2(183)	CPKLLPCLHTLCSGCLEASGMQCPICQAPWPLGADTPALDNVFFESLQR(3)-KTNNIFCSNPNHR(1)	3.22E-21	21	13.1
PML1(65)-PML2(65)	CQQCQAEAKCPK(9)-CQQCQAEAKCPK(9)	2.89E-07	7	33.6
PML1(65)-PML2(150)	CQQCQAEAKCPK(9)-ESADFWCFECEQLLCAKCFEAHQWFLK(17)	3.72E-03	2	34.2

*We used best E-value (1.00E-2) and Spec count of at least 2 as the thresholds to remove extra XL-MS data with lower confidence, and a Cα-Cα distance of less than 35 Å as the criterion to validate our cryo-EM model.

Table S4. XL-MS detected inter-subunit interactions of PML_{46-256} dimer*

Protein1(site)-protein2(site)	Peptides	Best E-value	Spec count
PML1(133)- PML2(160)	CKESADFWCFECEQLLCAK(2)-CFEAHQWFLKHEAR(10)	1.99E-07	2
PML1(160)- PML2(226)	CFEAHQWFLKHEAR(10)-PLCCSCALLDSSHSELKCDISAEIQQR(17)	6.49E-14	5
PML1(150)- PML2(183)	ESADFWCFECEQLLCAKCFEAHQWFLK(17)-KTNNIFCSNPNHR(1)	4.11E-13	4
PML1(209)- PML2(226)	GCSKPLCCSCALLDSSHSELK(4)-PLCCSCALLDSSHSELKCDISAEIQQR(17)	9.03E-18	13
PML1(183)- PML2(183)	KTNNIFCSNPNHR(1)-KTNNIFCSNPNHR(1)	1.86E-09	49
PML1(133)- PML2(183)	CKESADFWCFECEQLLCAK(2)-KTNNIFCSNPNHR(1)	3.78E-17	6
PML1(68)- PML2(183)	CPKLLPCLHTLCSGCLEASGMQCPICQAPWPLGADTPALDNVFFESLQR(3)-KTNNIFCSNPNHR(1)	2.95E-17	11
PML1(65)- PML2(65)	CQQCQAEAKCPK(9)-CQQCQAEAKCPK(9)	3.69E-07	6

*We used best E-value (1.00E-2) and Spec count of at least 2 as the thresholds to remove extra XL-MS data with lower confidence.

Table S5. XL-MS detected subunit interactions of the PML-CC domains*

Protein1(site)-protein2(site)	Peptides	Best E-value	Spec count
PML1(337)- PML2(183)	MKCYASDQEVLDMHGFLR(2)-KTNNIFCSNPNHR(1)	4.36E-18	21
PML1(328)- PML2(183)	KTNNIFCSNPNHR(1)-TGSALVQR(1)	4.26E-16	15
PML1(337)- PML2(65)	MKCYASDQEVLDMHGFLR(2)-CQQCQAEAKCPK(9)	1.97E-11	8
PML1(339)- PML2(183)	CYASDQEVLDMHGFLR(2)-KTNNIFCSNPNHR(1)	1.83E-23	3
PML1(328)- PML2(65)	CQQCQAEAKCPK(9)-TGSALVQR(1)	1.74E-21	8
PML1(328)- PML2(196)	TPTLTSIYCR(1)-TGSALVQR(1)	5.59E-06	4
PML1(328)- PML2(208)	GCSKPLCCSCALLDSSHSELK(3)-TGSALVQR(1)	7.63E-06	2
PML1(330)- PML2(183)	KTNNIFCSNPNHR(1)-TGSALVQR(3)	1.61E-06	2
PML1(328)- PML2(209)	GCSKPLCCSCALLDSSHSELK(4)-TGSALVQR(1)	1.30E-13	2

*We used best E-value (1.00E-2) and Spec count of at least 2 as the thresholds to remove extra XL-MS data with lower confidence.

References

1. Wang, P. et al. RING tetramerization is required for nuclear body biogenesis and PML sumoylation. *Nature Communications* **9**, 1277 (2018).
2. Li, Y. et al. B1 oligomerization regulates PML nuclear body biogenesis and leukemogenesis. *Nature Communications* **10**, 3789 (2019).
3. Bercier, P. et al. Structural Basis of PML-RARA Oncoprotein Targeting by Arsenic Unravels a Cysteine Rheostat Controlling PML Body Assembly and Function. *Cancer Discov.* **13**, 2548-2565 (2023).
